# Primer selection impacts the evaluation of microecological patterns in environmental microbiomes

**DOI:** 10.1002/imt2.135

**Published:** 2023-09-17

**Authors:** Jintao He, Tong Zhou, Xiaoqiang Shen, Nan Zhang, Chao Sun, Shipeng Lu, Yongqi Shao

**Affiliations:** ^1^ Max Planck Partner Group, Institute of Sericulture and Apiculture, Faculty of Agriculture, Life and Environmental Sciences Zhejiang University Hangzhou China; ^2^ Laboratory of Marine Organism Taxonomy and Phylogeny, Qingdao Key Laboratory of Marine Biodiversity and Conservation, Institute of Oceanology Chinese Academy of Sciences Qingdao China; ^3^ Analysis Center of Agrobiology and Environmental Sciences Zhejiang University Hangzhou China; ^4^ Institute of Botany Jiangsu Province and Chinese Academy of Sciences Nanjing China; ^5^ Key Laboratory of Silkworm and Bee Resource Utilization and Innovation of Zhejiang Province Hangzhou China; ^6^ Key Laboratory for Molecular Animal Nutrition Ministry of Education Hangzhou China

## Abstract

This study revealed that primer selection substantially influences the taxonomic and predicted functional composition and the characterization of microecological patterns, which was not alleviated by close‐reference clustering. Biases were relatively consistent across different habitats in community profiling but not in microecological patterns. These primer biases could be attributed to multiple aspects, including taxa specificity, regional hypervariability, and amplification efficiency.

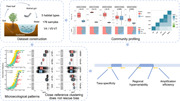

Investigations into microbial community composition offer extensive insights into the diversity, ecosystem function, and complex microbial processes [1, 2, 3]. Since the advent of high‐throughput sequencing, amplicon sequencing (e.g., 16S ribosomal RNA [rRNA] marker genes for prokaryotes) has been instrumental in the large‐scale monitoring of microbial communities across various habitats with high resolution and low cost [4, 5, 6]. In particular, unraveling microecological patterns, such as species abundance distribution (SAD), beta‐diversity, and assembly processes, can enhance our understanding of community characteristics and ecosystem functionality [7, 8, 9]. SAD, for example, is visually intuitive and easy to implement for quick assessments of ecosystem health and/or the success of management prescriptions to ameliorate the effects of disturbance [10]. Beta‐diversity provides insights into the mechanisms driving biodiversity loss and maintenance [11, 12]. Additionally, community assembly (determinism and stochasticity) has emerged as a focal point in microecology across diverse habitats, shedding light on the generation of extensive microbial diversity and its correlation with macroorganisms [13].

Despite the growing number of environmental microbiome studies, the validity of comparing results or data from different studies remains limited. One critical constraint is the application of various primer pairs. Over 60 universal primer pairs targeting bacterial 16S rRNA genes have been employed in 1 year across various habitats in amplicon sequencing‐based microecological studies (Table S1, 2021, details in Supporting Information) because of habitat type limitations, target species, continuous primer evaluation, the discovery of new species, and the improvement of database [14, 15]. The 515F/806R (V4) is recommended by the Earth Microbiome Project and is most commonly used. Nonetheless, primer pairs, such as 799F/1193R targeting V5–V7, were designed for minimizing the amplification of host organelle DNA, for example, chloroplast [16]. Researchers may also prefer certain primers in other habitats within their research project, which potentially exacerbates bias and limits data integration with other studies [17]. A typical example is 799F/1193R, which is not only used in plant endosphere but also in episphere, soil, and animal gut [18, 19, 20]. Previous attempts have been made to evaluate primer bias through in silico analysis and/or artificial community generation; however, these endeavors predominantly center on the assessment of taxa coverage and abundance estimation [21, 22, 23, 24]. To our knowledge, no studies have yet examined the impact of primer selection on microecological patterns across diverse habitats [25]. This knowledge gap impedes ecological interpretations pertaining to complex environmental microbiomes. Although close‐reference clustering (CR), a database‐dependent approach that maps recognizable sequences to a predefined set of reference sequences with known taxonomic information and discards unmapped sequences, is commonly employed in meta‐analyses to combine data sets generated from different primer sets [26, 27], its effectiveness in reducing primer bias remains unclear.

Mulberry‐dyke and Fish‐pond system (MF), which originated over 2500 years ago, is an important agricultural heritage ecosystem featuring internal material circulation as well as energy flow along the mulberry–silkworm–fish–sediment–soil [28]. MF provides a stable and controlled model to disclose primer effects across multiple habitat types, including terrestrial/aquatic and environment/plant/animal‐associated habitats [28]. Here, we examined bacterial communities across five different types of habitats in MF using the two primer pairs (V4: 515F/806R; V5–V7: 799F/1193R) mentioned above. In view of the foregoing, we focused on assessing the effect of primer selection on (1) the taxonomic composition and functional prediction in different habitats; (2) the microecological patterns including SAD, beta‐diversity, null model, normalized stochasticity ratio, neutral model, and so forth. By examining the same sample set in a controlled system, our study contributes to a better understanding of primer bias, and furthermore, its implication for interpreting microecological patterns among multiple habitats. Ultimately, these findings will inform better experimental design and data analysis strategies for more accurate and reliable insights into the ecological roles of microbial communities in various ecosystems.

## RESULTS AND DISCUSSION

### Primer bias in taxonomic composition

**Figure 1:**
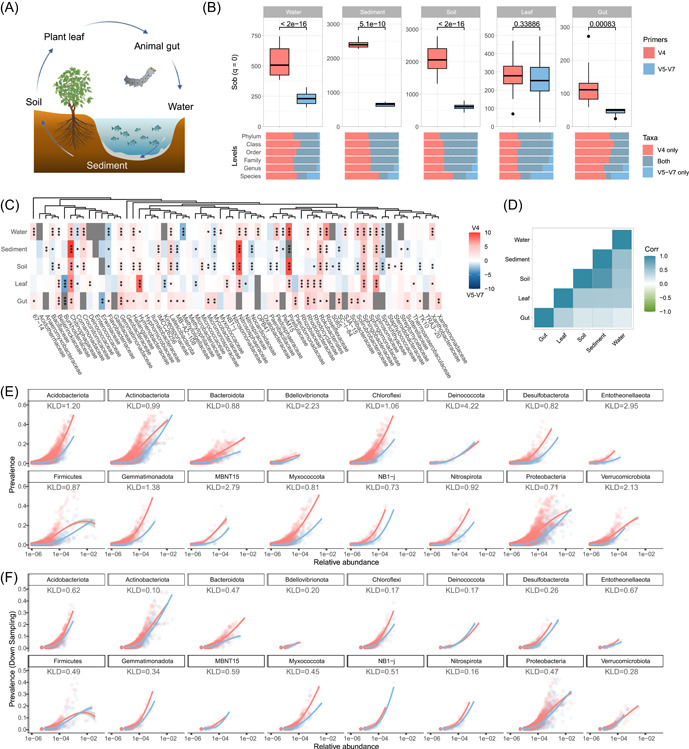
Primer bias in profiling bacterial community. (A) Overview of samples used in this study. A total of 176 biological samples (after quality control, sequence depth filtering, and sample pairing, 176 amplicon sequencing data in V4 and V5–V7, respectively) from five habitats, including water (*n* = 36) and sediment (*n* = 7) in fish‐pond, bulk soil (*n* = 79) and mulberry plant leaf (*n* = 40) in mulberry‐dyke, and silkworm gut (*n* = 12), were obtained from the Mulberry‐dyke and Fish‐pond system (MF). (B) Difference in the total number of species observed (Hill number *q* = 0, °D) of microbial communities in each habitat between different primers. Bottom panel represents the percentage of taxa that are shared and uniquely identified by primers at different levels. (C) Primer bias in the abundance of major taxonomic families in each habitat. Microbial groups were arranged according to their phylogenetic placement. Color represents the log 2‐fold ratio of abundance value of major families observed between two primer sets, indicating that taxon abundance is higher in V4 (red) or in V5–V7 (blue) data sets. Significances were calculated using the edgeR quasi‐likelihood test. (D) Consistency of primer bias in taxa abundance estimation across habitats. Color represents the correlation coefficients between the bias value vectors (log 2‐fold ratio of bias between two primer data sets in each taxon) in each habitat pair. High correlation coefficient represents that the primer bias in species abundance estimation was consistent between habitats. (E) The species abundance distribution (SAD) of amplicon sequence variants (ASVs) in major phyla. Each point represents an ASV. The line represents the Loess curve fit. The Kullback–Leibler Divergence (KLD) value represents the divergence of SAD between primer data sets. (F) SAD after down sampling. (*p ≤ 0.05, **p ≤ 0.01, ***p ≤ 0.001, and n.s. = *p* > 0.05.)

We sequenced V4 and V5–V7 regions of the 16S rRNA gene from the same sample set (Figure 1A), processed with the same approach for quality control, sequences denoising, and amplicon sequence variants (ASVs) resolving (Supporting Information) and found that alpha‐diversity, measured by Hill number diversity indices, varied significantly between primer sets, with V4 showing higher values in most habitats (Figure 1B and Supporting Information Figure S1A). Consistently, taxonomic annotation indicated that V4 exhibited superior performance in obtaining diverse taxa at various taxonomic levels (Figure 1B). This was further substantiated by the primer coverage evaluation and the taxonomic composition (Supporting Information Figures S1B and S2). Differential analyses revealed taxon‐dependent patterns biased by primers across habitats (Figure 1C). For instance, Chitinophagaceae, Sphingomonadaceae, Rokubacteriales, and Nitrososphaeraceae showed higher abundance in V4, whereas Nocardioidaceae, Comamonadaceae, and Burkholderiaceae were more abundant in V5–V7. The correlation tests employing the primer bias values of each taxon's abundance between habitats revealed distinct positive correlations, confirming that taxa overrepresented in specific habitats are more likely to be overrepresented in other habitats (Figure 1D).

To determine whether primer selection would impact the SAD pattern within different ecological subsets, we performed SAD analysis using ASVs from major phyla (Figure 1E). We found that V4 and V5–V7 generated similar but distinct SAD, as assessed by Kullback–Leibler Divergence. The differential patterns might be attributed to variations in taxa specificity and amplification efficiency [29, 30]. Two strategies were employed to investigate these divergences: the first strategy simulates the specificity of taxa coverage by using genera detectable by both primers; the second strategy simulates the amplification efficiency effects through in silico down sampling (Supporting Information). We found generally reduced divergence, pointing to their critical roles in shaping SAD (Figure 1F and Supporting Information Figure S3).

Our findings confirm the general influence of primer choice on diversity and composition characterization across habitats [31, 32]. Previous studies have reported superior performance of V4 primer in soil [25] but lower in leaf [32, 33]. Consistently, our results showed that V5–V7 are comparable to or outperform the V4 in leaf. Apart from species estimation, SAD was also affected by primer selection. These primer biases could be attributed to differential taxa specificity and amplification efficiency. Moreover, the regional information scale, for example, the longer region and higher regional hypervariability, can affect taxonomic resolution [34, 35, 36, 37], potentially explaining the improved taxa detection at the species level in V5–V7. These findings underscore the bias inherent in marker gene‐based metagenomics, resulting from the imbalances among primer sequences, amplification systems, and reaction condition optimization.

How can the primer bias in community profiling be minimized? Our results suggest the possibility of mitigating this bias through calibration across habitats, as evidenced by the correlation plot (Figure 1D). For instance, given that the abundance of Sphingomonadaceae was underrepresented in V5–V7, an appropriate calibration factor could be applied when integrating data from this region [38].

### Primer bias in functional prediction and beta‐diversity

**Figure 2:**
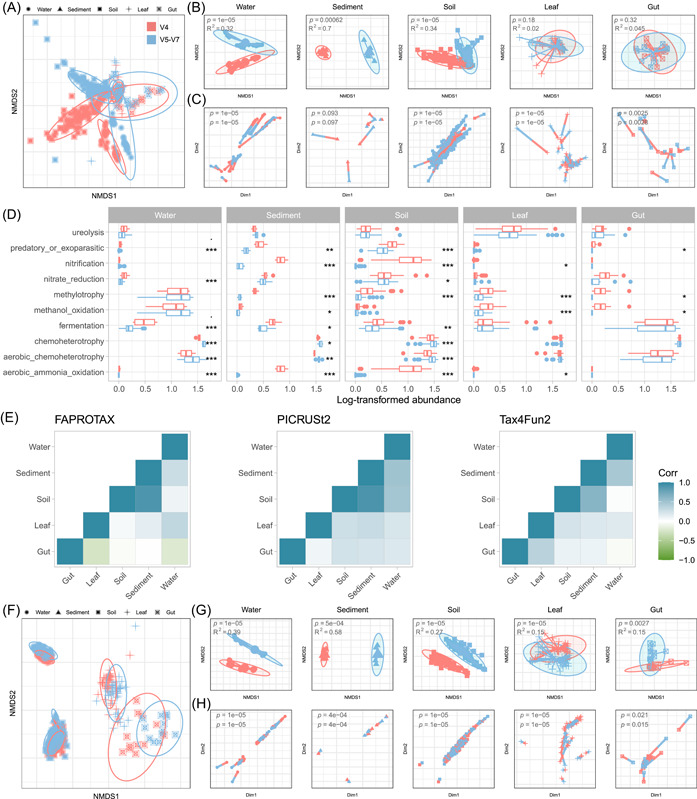
Primer bias in assessing function prediction and beta‐diversity. (A) Nonmetrix multidimensional scaling (NMDS) plot reflects primer differences in microbial predicted functional composition predicted by functional annotation of prokaryotic taxa (FAPROTAX). (B, C) NMDS plots and Procrustes plots of predicted functional composition between V4 and V5–V7 data sets in each habitat. The *R* ^2^ and *p* values from permutational multivariate analysis of variance compare community compositions characterized by different primers. In Procrustes plots, for a given sample, red lines connect to data from the V4 data set, while blue lines connect to points generated from the V5–V7 data set. The *p* values of Procrustes and Mantel tests are shown. (D) Differences of primer data sets in the abundances of major potential functions predicted by FAPROTAX (Welch *t*‐test). (E) Consistency of primer bias in functional composition across habitats based on FAPROTAX, Phylogenetic Investigation of Communities by Reconstruction of Unobserved States (PICRUSt2), and Tax4Fun2. Color represents the correlation coefficients between the bias value vectors (log 2‐fold ratio of bias between two primer data sets in each predicted function) in each habitat pair. High correlation coefficient represents that the primer bias in functional prediction was consistent between habitats. (F) NMDS plot reflects primer differences in microbial taxonomic composition at the genus level. (G, H) NMDS and Procrustes plots of taxonomic composition between V4 and V5–V7 data sets in each habitat.

The field of microecological study has witnessed extensive development in applications and software for functional prediction, yet little attention has been devoted to the potential impact of primer bias. We further performed functional annotation of prokaryotic taxa (FAPROTAX) to evaluate primer bias in functional prediction [39]. At the community level, predicted function profiles were significantly biased between primer data sets in most habitats, except for two host‐associated (leaf and gut) microbiotas (Figure 2A,B), but the distribution of samples was maintained (Figure 2C). Regarding the functional categories, most predicted functions (9 in 10) were affected considerably, which varied among habitats (Figure 2D), such as higher fermentation and aerobic ammonia oxidation in V4 data sets in water, sediment, and soil. Moreover, Phylogenetic Investigation of Communities by Reconstruction of Unobserved States (PICRUSt2) [40] and Tax4Fun2 [41] revealed similar results (Supporting Information Figure S4). A correlation test was used to examine the primer bias of each predicted function's abundance between habitats. The outcomes demonstrated positive correlations except for gut habitats in FAPROTAX and Tax4Fun2 prediction (Figure 2E). On the basis of predicted functions, we calculated the functional redundancy [42] within each community and found that primer selection can significantly affect inferred functional redundancy across most habitat types (Supporting Information Figure S4C). As functional prediction is derived from taxonomic composition, observed bias can result from primer bias in profiling specific functional taxa, for example, Nitrososphaeraceae. Intriguingly, functional prediction of two host‐associated (leaf and gut) microbiotas exhibited relatively lower levels of bias, potentially due to their distinct and less complicated taxonomic compositions. Hence, it is imperative to consider the influence of primer bias in functional prediction when integrating different primer data sets.

In terms of beta‐diversity, we found distinct clustering patterns based on habitat type and primer (Figure 2F), even when using genera shared between two primer data sets (Supporting Information Figure S5). The permutational multivariate analysis of variance indicated that the Habitat and Primer factors, as well as their interaction, significantly influenced community structures (*R* ^2^ = 0.597, 0.06, and 0.04, respectively, Supporting Information Table S2), suggesting a possible stronger primer bias in certain habitats, like, sediment (Figure 2G). As with the previous report [23], despite variations in sensitivity to diversity and taxonomic composition, different primers could capture similar intercommunity relationships (beta‐diversity) (Figure 2H), which may be attributed to the consistent bias of specific taxa across samples as evidenced above. These results imply that microecological patterns generated from different primers might be similar. However, unexpectedly, community variation quantification and beta‐diversity component partitioning varied between primers, such as lower similarity and higher nestedness components (Supporting Information Figure S6), likely due to differential taxa specificity and amplification through biased species abundance estimation. This could also explain the consistent discrepancy between primers in community variation quantification and beta‐diversity patterns across habitats. Collectively, these findings confirm that primer selection can subtly influence community structure and potentially introduce bias in microecological patterns.

### Primer bias in the characterization of microecological pattern

**Figure 3:**
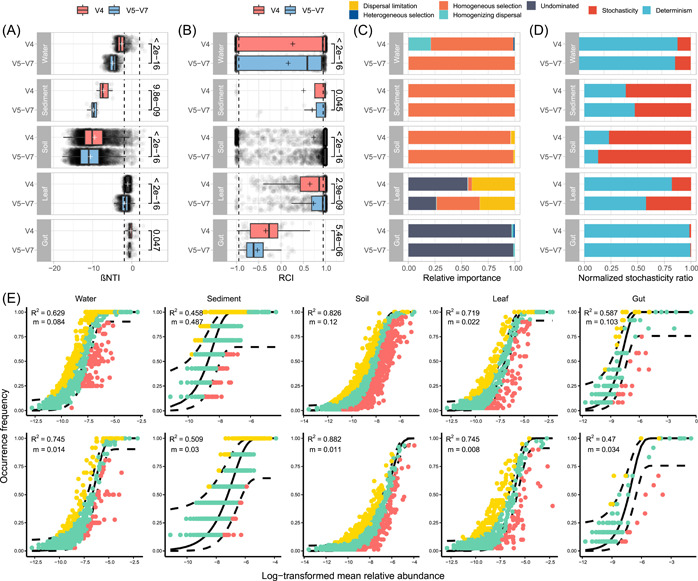
Primer bias in evaluating the community assembly process. STEN analysis based on βNTI (A) and RCI (B) for all pairwise community comparisons in each habitat in two primer data sets. Dashed lines at βNTI = −1.96 (homogeneous selection) and βNTI = 1.96 (variable selection) denote significance thresholds of phylogenetic signals. Dashed lines at RCI = −0.95 (homogenizing dispersal) and RCI = 0.95 (dispersal limitation) denote significance thresholds of taxonomic signals. Boxplots show the median (line), mean (plus sign), 25th and 75th percentiles (box), and 1.5× the interquartile range (whiskers). (C) Assembly process quantification: homogeneous selection (βNTI < −1.96; determinism), heterogeneous selection (βNTI > 1.96; determinism), homogeneous dispersal (|βNTI| < 1.96 and RCI > 0.95; stochasticity), dispersal limitation (|βNTI| < 1.96 and RCI < −0.95; stochasticity), and undominated processes (|βNTI| < 1.96 and |RCI| < 0.95, for example, weak selection, weak dispersal, diversification, and drift; stochasticity). (D) NST analysis quantifying taxonomic normalized stochasticity ratio in each habitat in two primer data sets. (E) SLON fitness indicated by *R* ^2^ values (fit to neutral assembly process) and m values (estimated migration rate) in each habitat in two primer data sets. Each point represents an amplicon sequence variant (ASV) colored based on the comparison of the actual taxon distribution (solid line) and 95% confidence interval (dashed lines) of model prediction, whether the ASV is above (yellow), below (red), or neutral (dark green). NST, Ning's normalized stochastic ratio model; RCI, Raup–Crick Index; SLON, Sloan's neutral model; STEN, Stegen's null model; βNTI, β‐nearest‐taxon index.

We evaluated primer bias in microecology community assembly by employing three widely used analytic frameworks: Stegen's null model (STEN), Ning's normalized stochastic ratio model (NST), and Sloan's neutral model (SLON). STEN framework was performed using null model‐based phylogenetic (β‐nearest‐taxon index, βNTI) and taxonomic diversity (modified Raup–Crick Index, RCI) sample pair metrics (Figure 3A,B). The βNTI revealed the significant effects of both habitats and primers (*p* < 0.001, analysis of variance) (Supporting Information Table S3), such as stronger homogeneous selection signals in V5–V7 (lower βNTI values). Furthermore, a significant interaction effect was observed between “Habitat ∗ Primer,” suggesting a stronger primer bias in certain habitats, such as sediment (*p* < 0.001, Supporting Information Table S3). Despite the RCI revealing similar results among primers, no consistent differences were found (Supporting Information Table S4). On the basis of calculated βNTI and RCI, we partitioned the assembly processes, revealing that some habitats in the V5–V7 data set exhibited greater relative importance of homogeneous selection, due to stronger phylogenetic signal captured by V5–V7 (Figure 3C). Second, NST showed deterministic processes in water, leaf, and gut and stochastic processes in sediment and soil for both primer data sets, with varying degrees between primers; for example, V4 exhibited more stochasticity in sediment but stronger determinism in soil and leaf than V5–V7 (Figure 3D). Primer bias was also observed in the SLON model, where *R* ^2^ fitness was lower in the gut but higher in other habitats, and the estimated migration rate (m) was higher in water but lower in the leaf and gut (Figure 3E). These observed primer biases were not alleviated by in silico down sampling (Supporting Information Figure S7). Moreover, community niche characterization, such as *B*com and *O*com, has been widely used [43, 44]. We found that *B*com was substantially influenced by primers, whereas *O*com exhibited relatively comparable between primers (Supporting Information Figure S8A). Additionally, primer bias was also found to affect co‐occurrence pattern characterization (Supporting Information Figure S8B,C).

To further corroborate our findings, we utilized another independent data set derived from four primer pairs, including V1–V2, V3–V4, V4, and V4–V5 [45]. The results consistently demonstrated a substantial impact of primer selection on microecological patterns (Supporting Information Figures S9, S10, and S11).

Microecological patterns are commonly employed to elucidate community assembly [46]. Our results revealed pronounced differences in assembly processes obtained from two primer data sets. Specifically, V5–V7 data set exhibited stronger phylogenetic signals than V4. These disparities may arise from the differential taxonomic composition yielded by differential resolution [34, 36]. The longer length and higher entropy of the V5–V7 region provide greater resolution than V4 [34], leading to divergent estimation of species relatedness/phylogenetic structure (lower βNTI) and community assembly process partitioning. In contrast, although RCI is also based on null models that compare observed communities with randomly expected ones, both community structure and null model expectation could be distorted by primer selection via biased taxa abundance estimation. This can account for the inconsistent primer bias across habitat types in RCI, NST, and SLON. Upon comparing different analysis methods, we noted that the response to primers varied significantly. While the impact of primers on *B*com was substantial, *O*com was less affected. These findings underscore that some analyses might not be suitable for data sets derived from different primers, even if they originate from the same biological sample set. We also observed discrepancies between analysis methods. For example, the gut habitat exhibited stochasticity in STEN but determinism in NST. This variation may arise from the different calculation methods (STEN considers both phylogenetic and taxonomic signals) and/or highly heterogeneous samples from different habitats, as varying group settings can alter NST values [47].

How can we minimize the primer bias in microecological patterns? Unfortunately, unlike taxa abundance, we did not observe consistent bias between different primer data sets in microecological patterns except for βNTI. These findings imply that the mechanism of primer bias in microecological patterns is intricate and operates at a fine scale, depending on specific environmental sample types. Previous studies have elucidated primer bias in species abundance estimation by comparing amplicon sequencing data with the known composition derived from mock communities [25, 48, 49]. Although the evaluation of microecological patterns is probably unsuitable for the mock community, other technologies such as less‐biased or gold‐standard shotgun sequencing hold promise in revealing the extent of absolute primer bias present in microecological pattern estimation.

### CR clustering does not reduce primer bias

Despite the broad application of CR clustering in meta‐analysis, few studies clarified the primer effect. By using Silva 138 [50] and Greengenes2 databases [51] on the same sample set, we found that CR discards an average of 26% of sequences, with variation depending on habitat type and database used (Supporting Information Figure S12). CR led to lower diversity due to the exclusion of unmapped sequences but yielded similar primer bias patterns in alpha‐diversity, SAD, taxa abundance, and beta‐diversity patterns (Supporting Information Figures S13 and S14). Furthermore, the relative importance of assembly processes in STEN, NST, and SLON fitness was also influenced, including increased homogeneous selection in leaf and gut habitats (Supporting Information Figure S15). Greengenes2‐based CR exhibited consistent trends (Supporting Information Figures S16, S17, and S18).

Our results are consistent with recent findings that demonstrated the strong effects of primer selection on CR‐based community profiling using machine learning [27]. Although amplicon sequences from different primers can be combined and aligned through representative sequence mapping, CR cannot fully address the biases between primers, such as information scale during sequence mapping and the over or underrepresentation of taxa due to taxa specificity and amplification bias [36]. Moreover, CR per se can influence the results and does not significantly reduce primer bias. These findings underscore the importance of caution when integrating data sets from different primers and suggest potential mitigation strategies, such as abundance calibration information scale correction and sample pairing [36, 52]. For example, a recent meta‐analysis integrated paired samples of bulk soils and rhizosphere from different studies, thereby mitigating the impact of primer bias [52].

Collectively, our analyses of these two typical primers highlight the bias between primers (i.e., relative bias) across habitats although within a specific agricultural ecosystem. Our findings suggest that the microecological patterns observed from various studies may partially result from primer bias. Nonetheless, the presence of primer bias does not preclude the comparison or integration of different primer data sets but emphasizes the need for greater attention, which should be given to the selection of primers used in amplicon data to mitigate primer bias. In terms of minimizing primer bias, we observed relatively consistent effects in community profiling among habitats, suggesting the potential for calibration to mitigate this bias, as described above. Nevertheless, it should be noted that complete calibration of taxonomic abundance estimations from different primers, particularly in microecological pattern evaluation, remains challenging. Thus, it is most advantageous to use an identical primer, like, V4, not only across different habitats but also in various studies, considering the wide array of primers being utilized. Although primers, such as 799F/1193R and 322F/796R, exhibited good performance in amplifying bacterial 16S rRNA genes from host total DNAs [53], the use of peptide nucleotide acid can be a valuable approach for blocking host amplification without introducing bias [54], allowing the broader application of V4 primers across different habitats and studies. Apart from relative bias between primers, to characterize the true diversity (minimizing absolute bias), researchers may consider primer sets with acceptable species coverage, and unbiased abundance estimation [34, 55]. For example, a recent study reported that V3–V4 is more similar to expected/true abundances based on standardized mock communities [48]. It is worth mentioning that while primer bias plays a critical role, biases in amplicon sequencing can also arise from other factors, including nucleic acid extraction, database bias, and sequencing platforms [21, 22]. Therefore, establishing general standards is essential to enhance the comparability of environmental microbiome profiling. Furthermore, the approaches employed here could be utilized to validate findings for universality in diverse natural habitats and in other meta‐barcoding‐based studies, for example, 16S rRNA gene for archaea, internal transcribed spacer for fungi, and 18S rRNA gene for micro‐eukaryotes [24, 49].

## CONCLUSION

This study constitutes a detailed analysis of the primer effects on both composition profiling and microecological patterns across various habitat types. Our findings reveal that primer selection not only influences diversity and composition estimations but also impacts functional prediction and microecological patterns, which could be attributed to multiple factors, including taxa specificity, regional hypervariability, and amplification efficiency. While biases in community profiling demonstrated a degree of consistency across varying habitats, they were less consistent in microecological patterns. These compelling findings raise a cautionary note, suggesting that the variance observed in microecological patterns across disparate studies may partially stem from the underappreciated influence of primer bias. Moreover, CR‐based analyses could not reduce such primer bias. To enhance the reliability and accuracy of ecological interpretation, further considerations and measures need to be taken to recognize and reduce primer bias.

## AUTHOR CONTRIBUTIONS

**Jintao He**: Conceptualization, methodology, and writing—original draft. **Tong Zhou**: Methodology, and writing—review and editing. **Xiaoqiang Shen**: Methodology. **Nan Zhang**: Methodology. **Chao Sun**: Investigation. **Shipeng Lu**: Investigation. **Yongqi Shao**: Supervision, funding acquisition, and writing—review and editing.

## CONFLICT OF INTEREST STATEMENT

The authors declare no conflict of interest.

## Supporting information

Supporting information.

Supporting information.

## Data Availability

All raw sequences from this study have been deposited in the public NCBI Sequence Read Archive (SRA) with the accession number PRJNA881590 (SRR21617369–SRR21617820, https://www.ncbi.nlm.nih.gov/bioproject/?term=PRJNA881590). The code scripts used for analysis and visualization have been deposited on GitHub (https://github.com/kingtom2016/primer_compare). Supplementary materials (figures, tables, scripts, graphical abstract, slides, videos, Chinese translated version, and updated materials) may be found in the online DOI or iMeta Science http://www.imeta.science/.
