# Supplementary material for: Primer selection impacts the evaluation of microecological patterns in environmental microbiomes
Source: Imeta. 2023 Sep 17;2(4):e135. doi: 10.1002/imt2.135 (PMC10989904; doi:10.1002/imt2.135)
Supplement: Supplementary file 1 — Supporting information. [file IMT2-2-e135-s001.docx]

*Supporting information for*

**Primer selection impacts the evaluation of microecological patterns in environmental microbiomes**

**Running title:**

Primer selection impacts microecological pattern evaluation

Jintao He^1^, Tong Zhou^2^, Xiaoqiang Shen^1^, Nan Zhang^1^, Chao Sun^3^, Shipeng Lu^4^, Yongqi Shao^1, 5, 6^*

^1^Max Planck Partner Group, Institute of Sericulture and Apiculture, Faculty of Agriculture, Life and Environmental Sciences, Zhejiang University, Hangzhou, China;

^2^Laboratory of Marine Organism Taxonomy and Phylogeny, Qingdao Key Laboratory of Marine Biodiversity and Conservation, Institute of Oceanology, Chinese Academy of Sciences, Qingdao, China;

^3^Analysis Center of Agrobiology and Environmental Sciences, Zhejiang University, Hangzhou, China;

^4^Institute of Botany, Jiangsu Province and Chinese Academy of Sciences, Nanjing, China;

^5^Key Laboratory of Silkworm and Bee Resource Utilization and Innovation of Zhejiang Province, Hangzhou, China;

^6^Key Laboratory for Molecular Animal Nutrition, Ministry of Education, Hangzhou, China

*Correspondence: Yongqi Shao, Fax: +86-571-88982757; E-mail: yshao@zju.edu.cn

**This Word file includes:**

Figure S1 to S18

Supporting Methods

References

**Other Supplementary Material for this manuscript includes:**

Tables S1 to S4 as a separate Excel file

# Supporting Figures

1. The differential alpha diversity and taxonomic composition between primer datasets. (A) The alpha diversity measured by Shannon entropy, Inverse Simpson index and Evenness between V4 and V5−V7 datasets in each habitat. (B) The phylum-level taxonomic composition between V4 and V5−V7 datasets in each habitat.

1. *In silico* evaluation of primer pairs at the phylum level. Bar represents the coverage for each taxonomic group against all of the taxonomies offered by SILVA 138.1 using TestPrime 1.0 with one (A) and two (B) mismatch allowed.


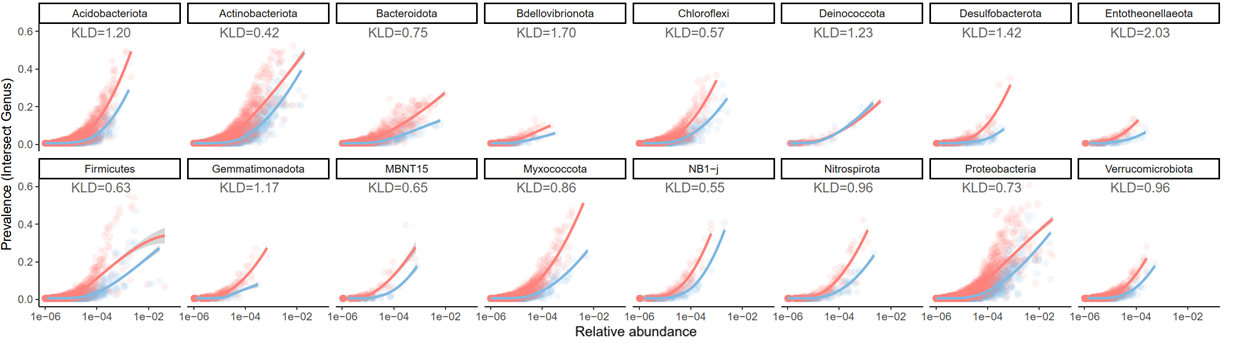


1. The species abundance distribution of ASVs in top abundant phyla using ASV belonging to genera that are shared between V4 and V5−V7 datasets. Color represents primers V4 (red) and V5−V7 (blue). The Kullback–Leibler Divergence (KLD) value represents the divergence of SAD between primer datasets.

1. Primer bias in assessing function prediction. Biased functional prediction based on PICRUSt2 (A) and Tax4Fun2 (B). (C) Primer bias in the estimation of functional redundancy. The *p*-values were shown (t-test, false discovery rate correction).


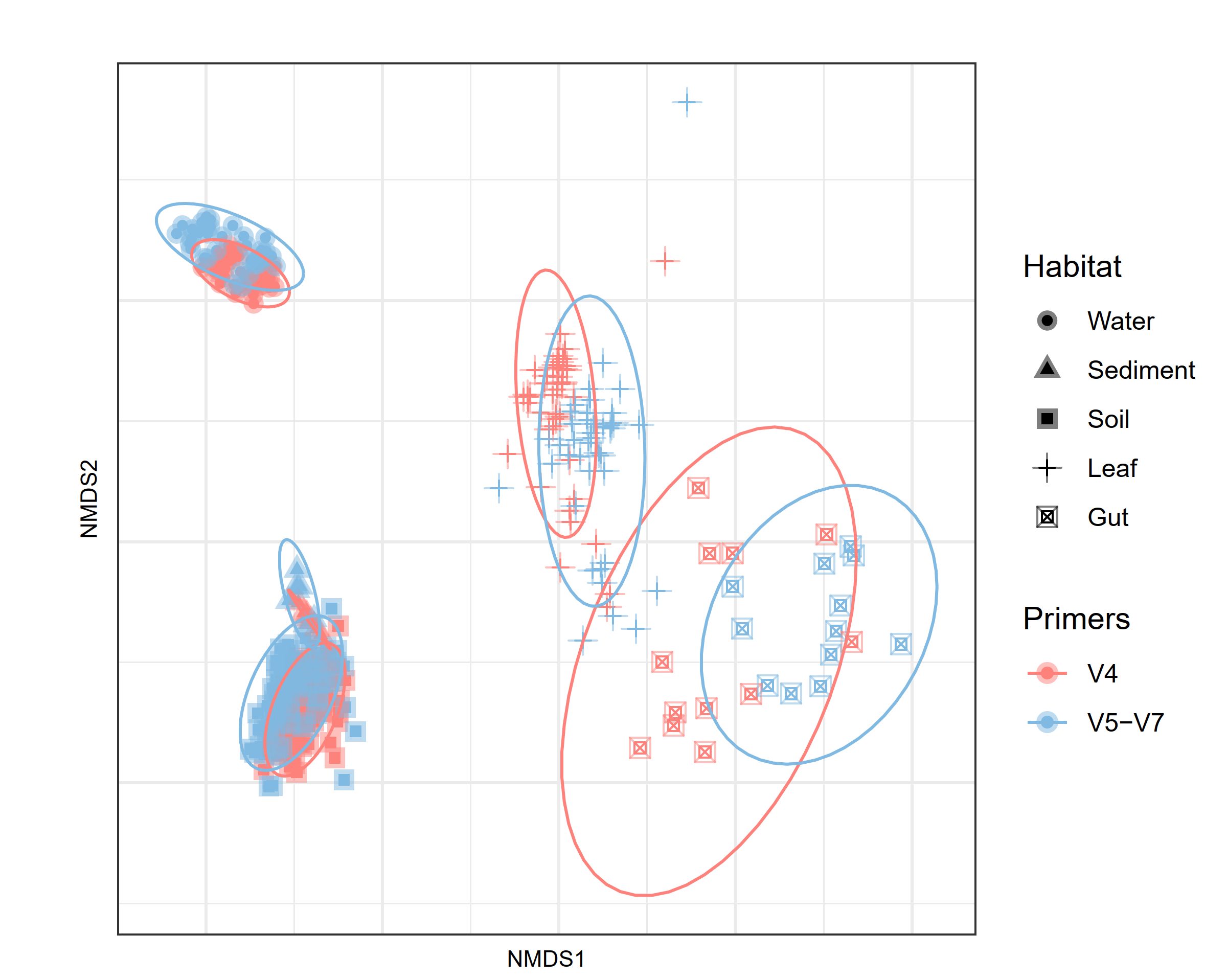


1. NMDS plots reflecting differences in microbial community composition of all habitats together, using the genera shared between two primer datasets.

1. Primer bias in the quantification of community variation and beta-diversity pattern. (left panel) Bar plots reflecting differences between primers in explaining the variation of bacterial composition amongst communities using ADONIS based on Bray–Curtis and Jaccard distances (taxonomic metrics) and weighted and unweighted UniFrac distance (phylogenetic metrics). Inter-Site (traditional and modern MF); Inter-Habitat (water, sediment, soil, leaf, and gut). (middle and right panel) Difference between primers in community beta-diversity partitioning using POD and SET frameworks, based on Sørensen dissimilarity. POD separates the total diversity of pairs of sites into three components, including Similarity (some species are present in both communities), Replacement (Repl, same number of species disappear in the first community and appear in the second community), and Richness difference (RichDif, two communities are not equal in species number); SET separates total diversity into Similarity, Nestedness (subsets of species from the richer site), and Intersection of nestedness and beta-diversity (INBD).


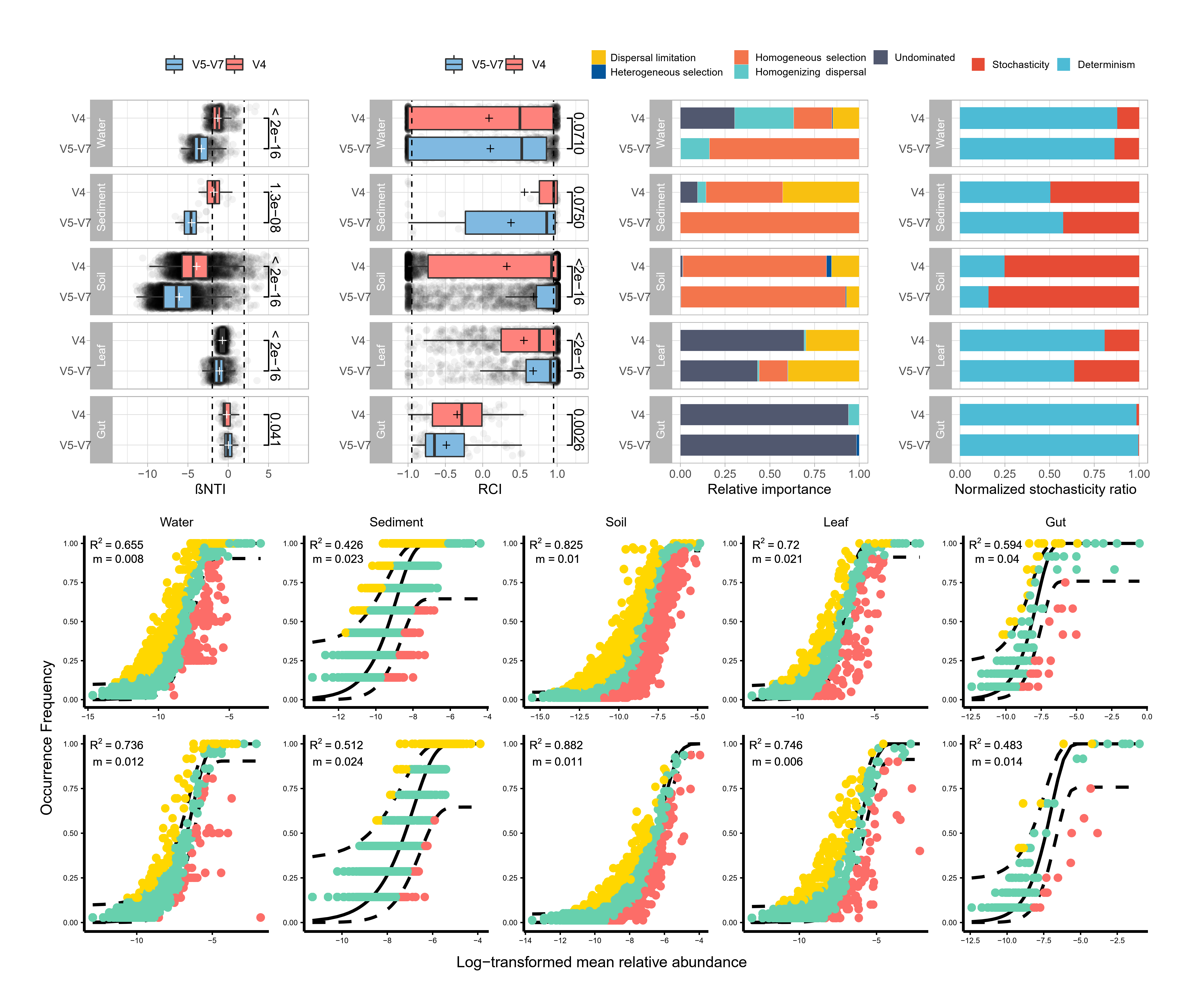


1. Primer bias in assessing the bacterial community assembly process based on data after down sampling.

1. Primer bias in niche characterization and co-occurrence patterns. (A) Habitat niche breadth (*B*com) and overlap (*O*com) inferred from V4 and V5−V7 datasets in each habitat type. (B) Topological properties of co-occurrence networks constructed from V4 and V5−V7 datasets in each habitat type. (C) C-score metrics in different habitats based on different primers. Gray and blue bars represent observed C-score and simulated C-score. Red points represent the standardized effect size. The values of observed C-score simulated C-score indicate non-random co-occurrence patterns. Standardized effect size <− 2 and > 2 represent aggregation and segregation, respectively.


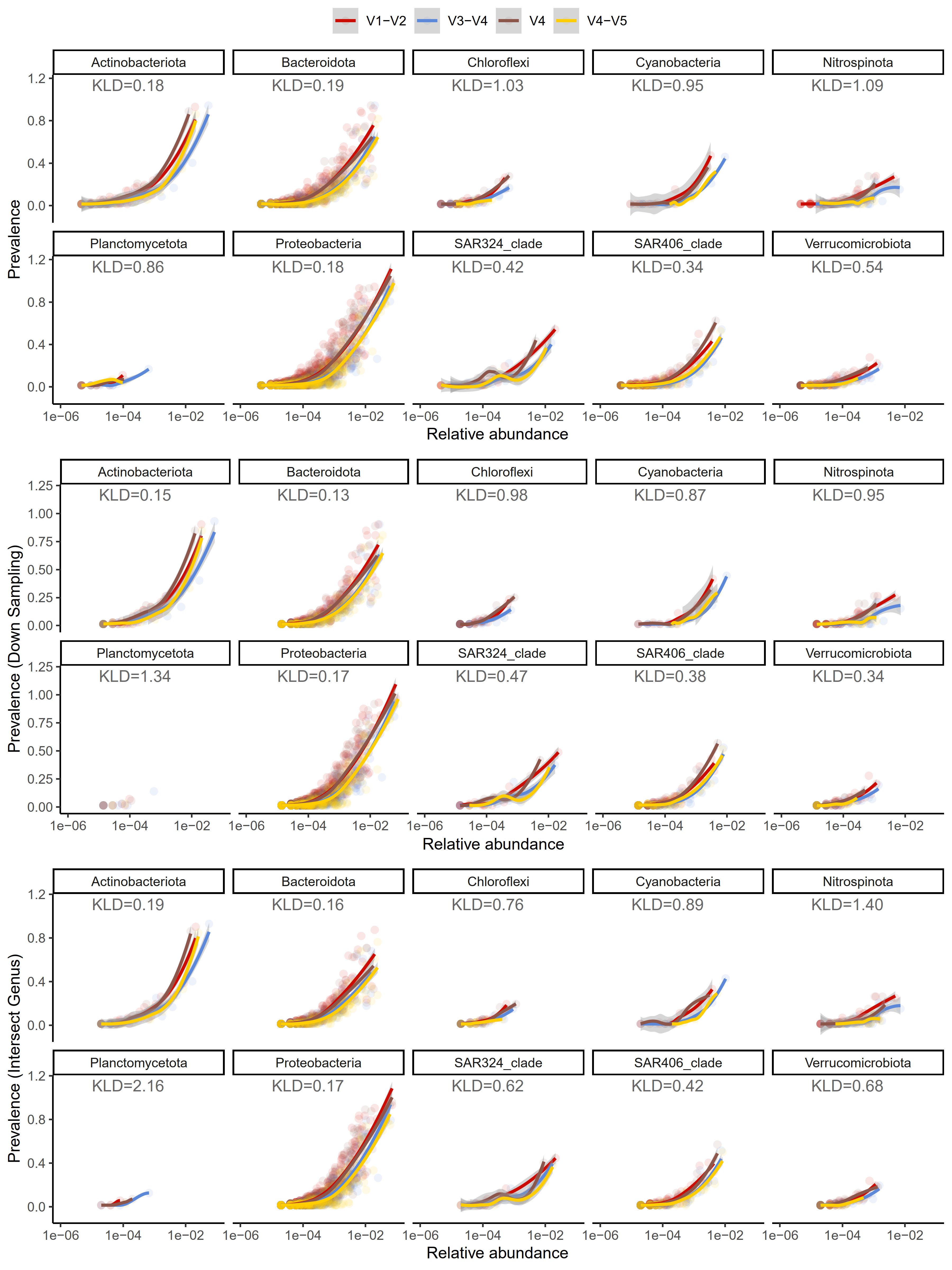


1. Primer bias in SAD using datasets generated from four primer sets. Colors represent different primer sets. The average KLD value between primer sets calculated in each phylum represents the SAD divergence.

1. Primer bias in characterizing the beta-diversity using datasets generated from four primer sets. The analysis was performed on communities at different depths including surface, 30m, 75m, 150m, and 300m.


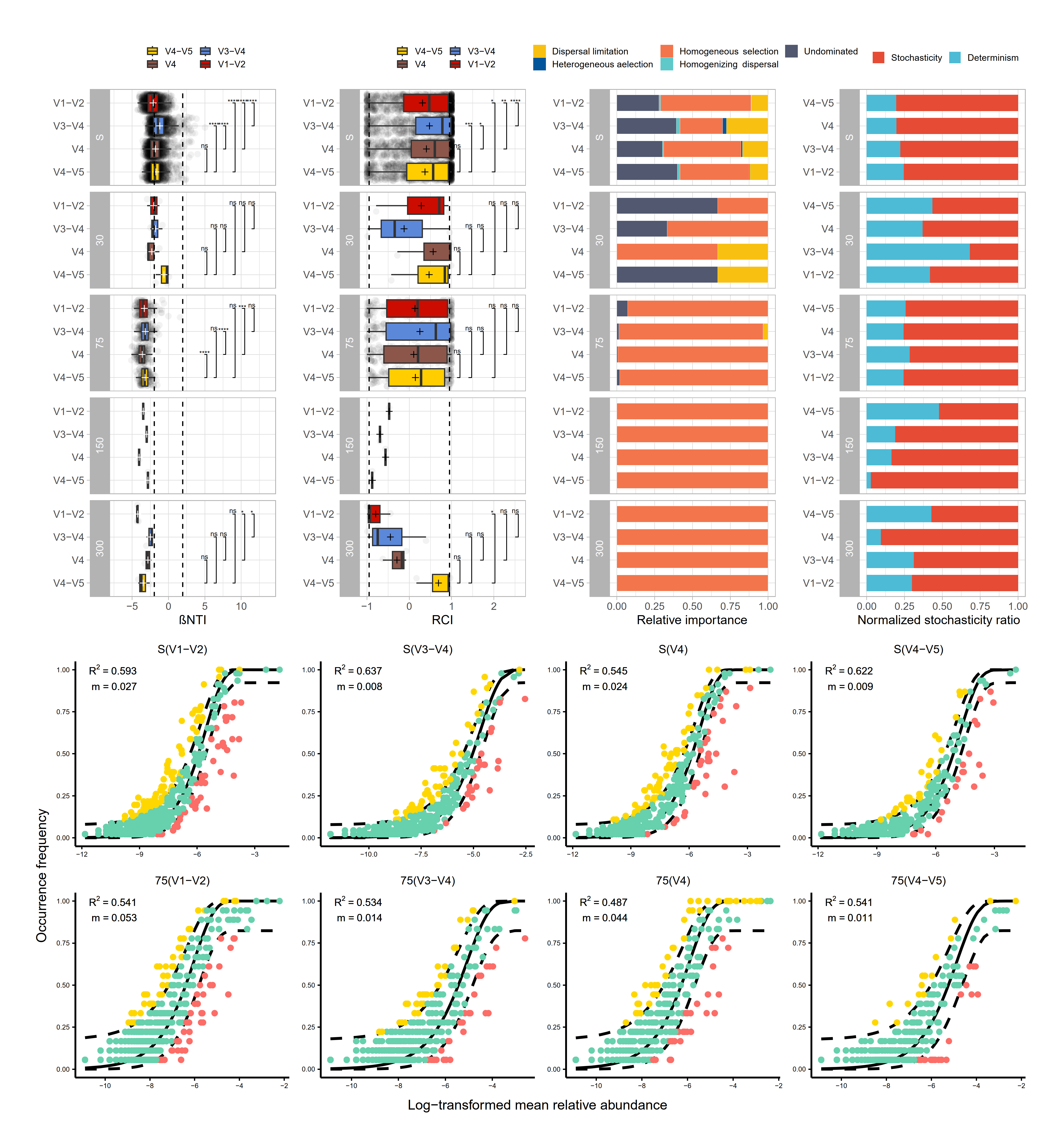


1. Primer bias in evaluating the community assembly process using datasets generated from four primer sets.


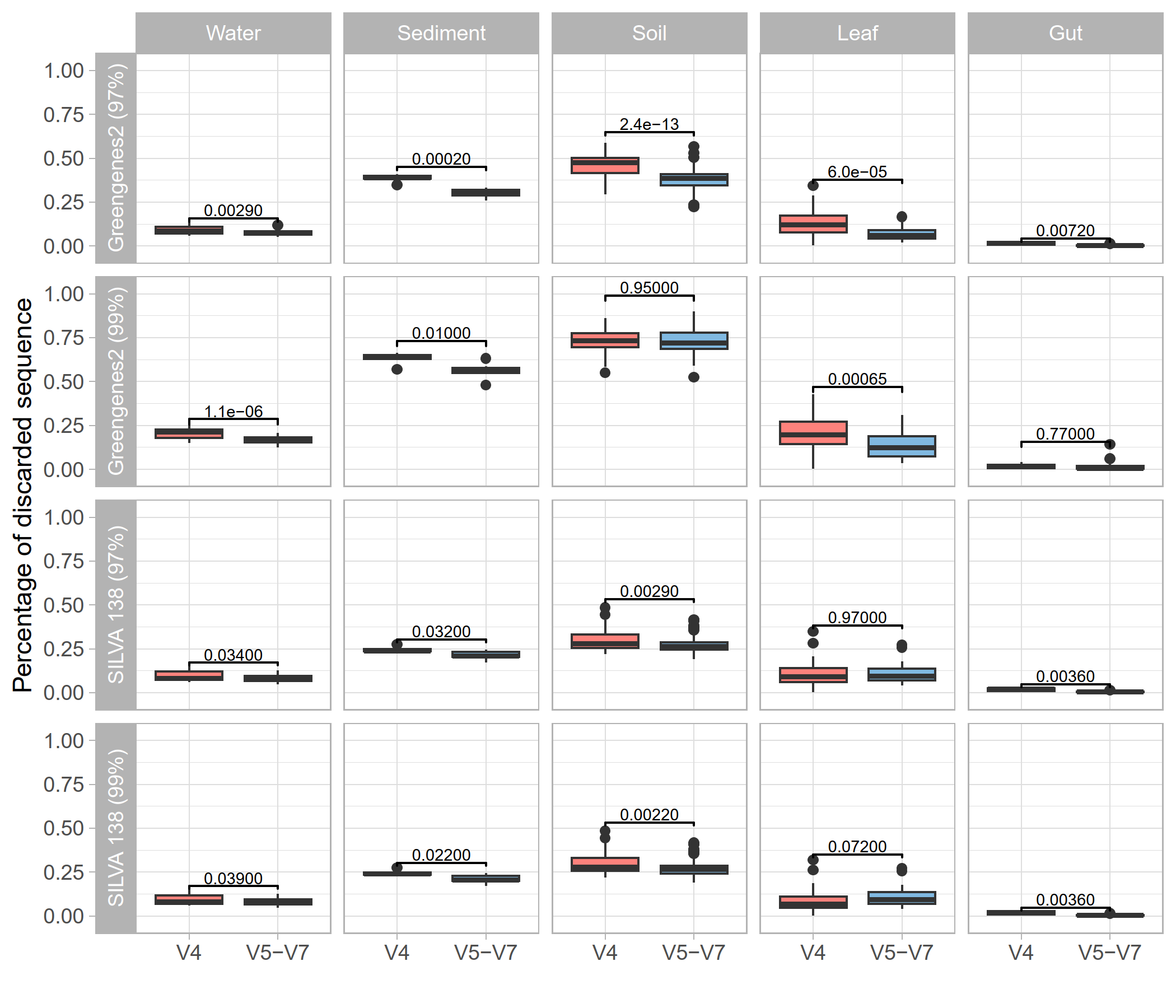


1. The percentage of sequences discarded during CR against Silva 138 and Greengenes2 database with the identity cutoff at 0.97 and 0.99. The *p*-values were adjusted with false discovery rate correction.

1. Primer bias in profiling bacterial community using CR with Silva 138 database.

1. Primer bias in characterizing the beta-diversity using CR with Silva 138 database.


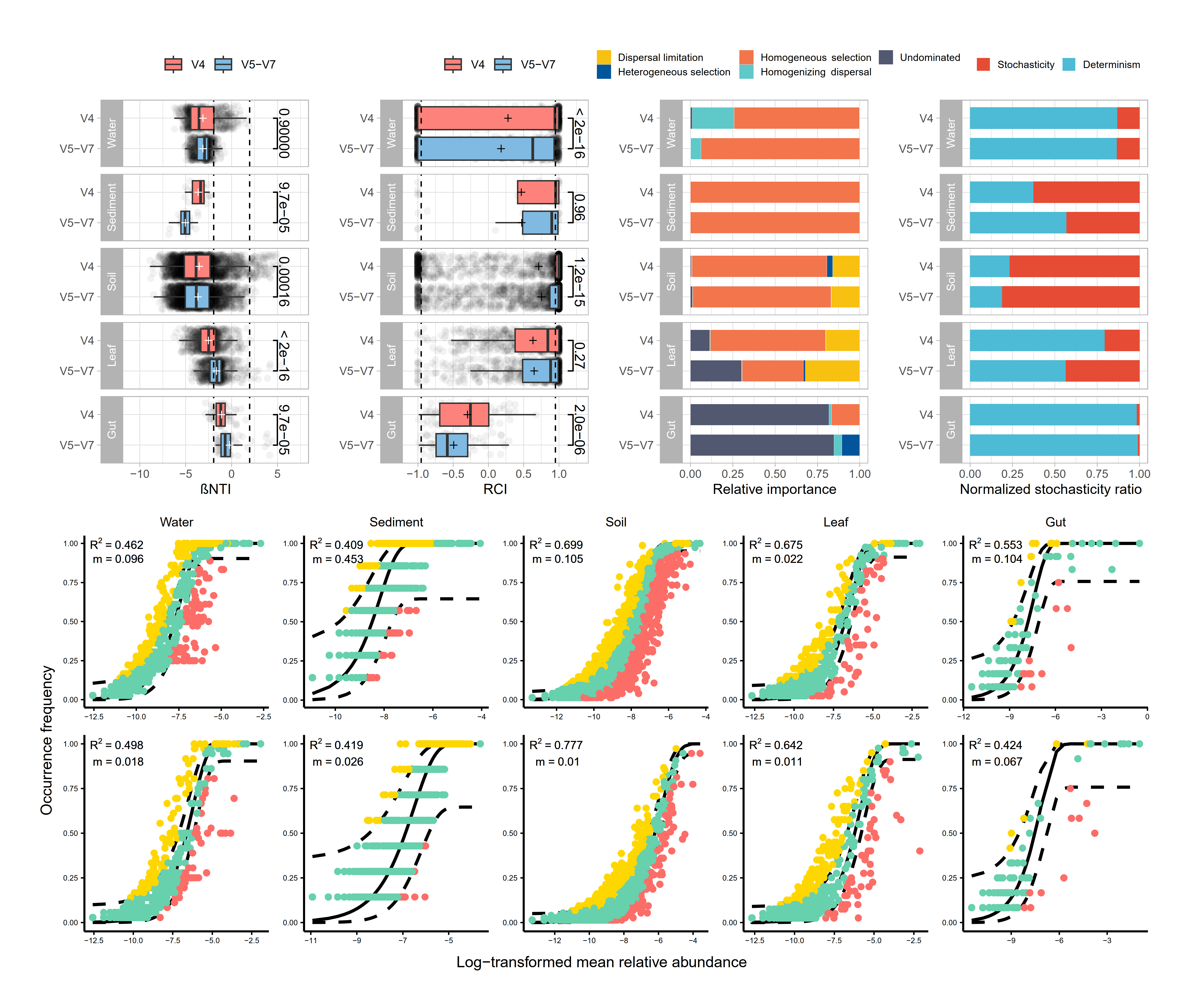


1. Primer bias in evaluating the community assembly process using CR with Silva 138 database.

1. Primer bias in profiling bacterial community using CR with Greengenes2 database.

1. Primer bias in characterizing the beta-diversity using CR with Greengenes2 database.


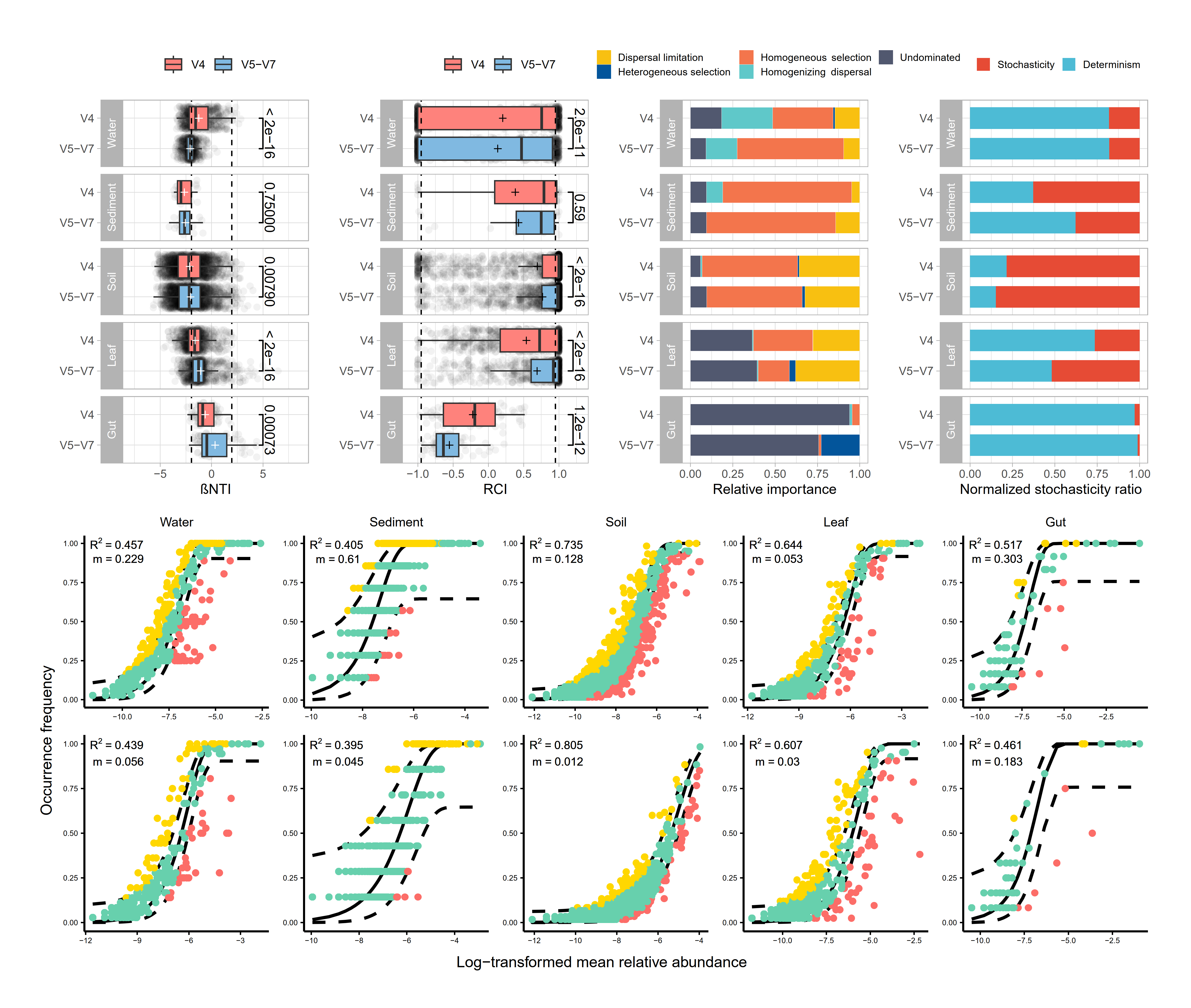


1. Primer bias in evaluating the community assembly process using CR with Greengenes2 database.

# Supporting Methods

**Literature search and sample collection**

We retrieved information on primer pairs (primers hereafter) used in microecological research based on the Web of Science Core Collection database from Jan. 2021 to Dec. 2021). Associated studies (amplicon sequencing targeting the bacterial community in environmental and plant microbiome) were retrieved using the search string: “((ALL=(environmental microbiome 16S rRNA gene sequencing)) OR (ALL=(plant microbiome 16S rRNA gene sequencing))) AND (DT==(“ARTICLE”) AND PY==(“2021”))”. Research lists were manually screened to identify primers used for amplicon sequencing. Amongst all retrieved research (more than 200), approximately 8.6% did not provide information about the names, sequences, or references of applied primers. 515F/806R-related primers (V4) were commonly used, whereas 799F-related primers were frequently used in multiple habitats, e.g., plant endosphere, episphere, and soil microbiota, in studies.

Samples were collected from two MF sites in the Globally Important Agricultural Heritage Systems (GIAHS) in Huzhou, Zhejiang Province, China (30.7014 N, 120.1320 E) [1]. The MF is a compound agroecosystem composed of several subsystems such as fish pond, mulberry dyke, and silkworm. Five habitat types (water, sediment, soil, plant leaf, and animal gut) were chosen due to their essential roles in MF, microbial resource, research, and biosphere [2−4]. By internal material circulation and energy flow, the balance and coordination between economic benefits and ecological functions could be realized, contributing to relatively higher anti-interference and stability [4], thus providing a good model ecosystem for cross-habitat study. Modern MF is a recently developed in-pond [raceway](https://www.sciencedirect.com/topics/agricultural-and-biological-sciences/raceways) system as a sustainable pond farming approach that effectively reduces pollution and facilitates management [5] as the other site for sample collection. All samples were collected in Mar. 2021 and May 2021. Briefly, for water samples, a volume of 400 mL each of the collected water was ﬁltered by a 0.22 µm nylon filter (Millipore, USA). The ﬁlters were stored at -80°C until DNA extraction; Sediment samples were collected in replicate within a radius of 1 m and pooled together to yield a composite sample from each site [6]; Soil samples at the depths of 10 cm and 50 cm were collected as previously described [7]. Soil samples from different depths were combined in the following analyses; Fresh mulberry leaves were collected with three replicates from each plant. The total microbiotas of mulberry leaves, including epiphytes and endophytes, were used to represent microbiotas in the leaf habitat [2]; The silkworm hybrid strain “Mercury 1” was provided by Huzhou Academy of Agricultural Sciences. Silkworms were reared using fresh mulberry leaves collected from MF, and the whole gut of larvae on the second day of the fifth instar was collected [8]. All samples were transported to the laboratory on ice and processed for DNA extraction.

**Amplicon sequencing**

Genomic DNA was extracted from 250 mg of each sample using DNeasy PowerSoil Kit (QIAGEN, Germany) [9]. Samples homogenization was performed in a Precellys-24 tissue homogenizer (Bertin Technologies, France) at 5500 rpm for 60 s. The final DNA concentration and purity were determined by NanoDrop 2000 UV-vis spectrophotometer (Thermo Scientific, USA), and DNA quality was checked by 1% agarose gel electrophoresis. The 16S rRNA gene ampliﬁcation procedures were examined for each sample using 515F/806R (V4; GTGYCAGCMGCCGCGGTAA/ GGACTACNVGGGTWTCTAAT) and 799F/1193R (V5−V7; AACMGGATTAGATACCCKG/ ACGTCATCCCCACCTTCC), respectively (separate DNA of each biological sample into two parts. one for V4 and the other for V5−V7), as previously described [10,11]. For V4 amplification, we added a mixture of peptide nucleotide acid (PNA) blockers oligos (PNA Bio, USA) to inhibit the amplification of mitochondrial (mPNA) and chloroplast (pPNA) 16S rRNA genes without biasing results [12]. The resulting PCR products were extracted from a 2% agarose gel and purified using the AxyPrep DNA Gel Extraction Kit (Axygen Biosciences, USA) and quantified using QuantiFluor™-ST (Promega, USA) according to the manufacturer’s protocol. Purified PCR products were pooled in equimolar and paired-end sequenced (2 × 250) on an Illumina Novaseq platform (Illumina, USA). Notably, template-free water blanks were processed with the same DNA extraction and PCR ampliﬁcation kits as the negative controls to check for reagent and laboratory contamination, as described previously [2]. Two amplicon datasets generated using different primers were processed with the same approach to generate and annotate amplicon sequence variants (ASVs). Briefly, spurious sequences and primers were trimmed using Cutadapt (v2.8) [13], and the resulting sequences were merged using PEAR (v0.9.11) [14]. High-quality sequences were then denoised using the q2-dada2 plugin with default parameter to differentiate the partial 16S rRNA gene ASVs and to remove chimeras [15]. Taxonomic assignment was performed using a Naïve Bayes consensus taxonomy classifier pretrained on the SILVA (v138) databases [16]. ASVs classified as singletons, chloroplasts-assigned, and mitochondria-assigned were removed. Potential contaminant reads were identified using the decontam (v1.18.0) [17]. Only samples with sufficient read numbers (>10000) both in V4 and V5−V7 datasets were used. Finally, a total of 176 biological samples (176 amplicon data in the V4 dataset and V5−V7 dataset, respectively) were used for the following analyses, including water (n = 36) and sediment (n = 7), soil (n = 79), leaf (n = 40), and silkworm gut (n = 12), were obtained. Three function prediction pipelines, including Functional Annotation of Prokaryotic Taxa (FAPROTAX, v1.2.4), Phylogenetic Investigation of Communities by Reconstruction of Unobserved States (PICRUSt2, v2.4.1), and Tax4Fun2 (v1.1.5), were implemented in default settings to predict and compare community functions between primers [18−20]. The functional redundancy [21] was calculated within each community based on predicted functions from PICRUSt2.

**Beta-diversity pattern**

Beta-diversity measures compositional heterogeneity across communities (between-sample diversity), and it can be partitioned into species replacement and richness differences, enabling us to estimate the processes that produce beta-diversity patterns, such as similarity, replacement, richness differences, and nestedness. Similarity means that some species are present in both communities. Species replacement (same number of species disappear in the first community and appear in the second community) indicates the turnover of species amongst communities–for example–due to environmental filtering or competition [22]. Richness differences (two communities are not equal in species number) may reflect the different coexistence parameters in different locations and/or dispersal limitations independently of species replacement [23]. Nestedness is a type of richness difference characterized by subsets of species from the richer site. For beta-diversity partitioning, we applied two analytic frameworks, Podani’s framework (POD) and Set theory-based framework (SET) using Sørensen and Bray-Curtis dissimilarity index as described previously [23,24]. The definition, features, and differences of several beta-diversity partitioning frameworks, such as Baselga’s framework (BAS), POD, and SET have been documented [23,25,26]. Briefly, both POD and SET partitioning frameworks partition beta diversity into components and both rely on PPCs. The difference between them stems from the phenomenon they reflect. The components of the POD framework illustrate the ecological concepts of replacement and richness difference. In contrast, the SET framework reflects the intersection of nestedness and beta diversity and the relative complement of nestedness in beta diversity. These concepts, however, are manifested by the Replacement and Richness difference pairwise pattern components (PPCs) in a context-dependent way, due to the complex nature of the ecological definition of nestedness [23].

**Community assembly characterization**

Community assembly includes two fundamental types of process: Deterministic process, a niche-based process that shapes non-random community structure; Stochastic process, species coexist or overlap but do not eliminate one another since their competitive abilities are closely balanced, and their abundances thus randomly change with chance fluctuations [27].

Three state-of-the-art analytic frameworks were introduced to characterize the microbial community assembly processes, including Stegen’s null model (STEN), Ning’s normalized stochastic ratio model (NST), and Sloan’s neutral model (SLON) as described previously [28−30]. In STEN, the turnover of phylogenetic composition between samples (phylogenetic β-diversity) was first quantified using between-community Mean Nearest Taxon Distance (βMNTD) metric [31], as follows:

$\beta MNTD=0.5\left[ \sum_{i_{k}=1}^{n_{k}} f_{i_{k}}\min\left( \Delta i_{k}j_{m} \right)+\sum_{i_{m}=1}^{n_{m}} f_{i_{m}}min(\Delta i_{m}j_{k}) \right]$,

where $f_{i_{k}}$ is the relative abundance of species $i$ (ASV or OTU in our cases) in community $k$, $n_{k}$ is the number of species in $k$, and $\min\left( \Delta i_{k}j_{m} \right)$ is the minimum phylogenetic distance between species $i$ in community $k$ and all species $j$ in community $m$. βMNTD was calculated using the package “picante” with parameters as previously described [32]. The phylogenetic signal (signiﬁcance) was evaluated via the β-Nearest Taxon Index (βNTI) by comparing the observed βMNTD and the null model-based βMNTD, as follows:

$\beta NTI= \left( {\beta MNTD}_{\mathrm{obs}}-\bar{{\beta MNTD}_{\mathrm{null}}} \right)/{\mathrm{sd}\left( {\beta MNTD}_{\mathrm{null}} \right)}$,

where βMNTD_obs_ is observed βMNTD, null model-based βMNTD_null_ is calculated based on the same regional species pool by randomly shuﬄing species across the tips of the phylogeny, and sd indicates the standard deviation of the βMNTD_null_ distribution.

Similarly, the deviation in taxonomic diversity was characterized by Bray–Curtis dissimilarity-based Raup-Crick index (RCI). Assembly process partitioning was performed by STEN based on βNTI and RCI. A significant deviation in βNTI indicated the dominance of homogeneous or heterogeneous selection processes (i.e., βNTI < -2 or βNTI > 2, respectively). The RCI was then used to further partition those not assigned to selection (i.e., |βNTI| < 2). The relative importance of homogenizing dispersal and dispersal limitation was quantified as the fractions with RCI < -0.95 and RCI > 0.95, respectively. In NST, the importance of stochastic effects in the process of community assembly (ranging from 0 to 1) was measured using the taxonomic normalized stochasticity ratio (NST) with 50% as the boundary point, that is, whether the assembly was more deterministic (<50%) or more stochastic (>50%) processes [29].

In SLON, neutral model analysis was performed to evaluate the contribution of neutral/niche-based processes in microbial community assembly [30]. Since rare taxa in a metacommunity are likely to be lost from the individual community due to ecological drift, abundant taxa are generally predicted to be more widespread [33]. The parameter R^2^ predicts the overall ﬁt to the neutral model. The estimated migration rate (m) is the probability of species dispersal from the metacommunity to replace a randomly lost individual in the local community.

To provide further validation of our findings, we employed an independent dataset derived from a separate study [34]. This dataset was generated using four distinct primer pairs: V1-V2, V3-V4, V4, and V4-V5. Our method of microecological pattern analysis followed the same procedure applied to our primary dataset.

**Close-reference clustering**

A close-reference clustering (CR) approach was used to simulate the data integration in meta-analysis as previously described [35]. This approach is advantageous for comparing research that employs various 16S rRNA or ITS gene regions since the underlying database documents full-length sequences. Brieﬂy, the q2-vsearch plugin was used to map the above-obtained fragments to non-redundant full-length 16S rRNA sequences from the Silva (v138) [36] and Greengenes2 (v2022.10) [37] databases. Since CR discarded the unmapped sequences, we rarefied the CR datasets of Silva and Greengenes2 to 8000 and 3000, respectively.

**Statistical analysis**

All analyses were conducted in R 4.1.2. Normal distribution and homoscedasticity were assessed by Shapiro–Wilk and Levene’s tests, respectively. Hill number diversity indices (D) were calculated with the parameter q from 0 to 2. It is a mathematically uniﬁed parametric family of diversity indexes with a parameter (q), which determines the index’s sensitivity to species relative abundances [38]. Especially, when q = 0, D corresponds to species richness, when q = 1, D tends to the Shannon entropy, and when q = 2, D corresponds to the inverse Simpson index. A higher q will give more weight to the abundant species. TestPrime 1.0 [39] was used to perform *in silico* evaluation to investigate how well certain primer pairs align to microbes in the SILVA database. The primer pairs V4 and V5−V7 were compared to sequences found within the SSU r138.1 SILVA database. A Pearson’s correlation coefficient was calculated from two vectors of the taxa’s primer bias (changes in relative abundances) in each habitat pair at the family level. Differential abundance analyses were performed using the edgeR quasi-likelihood test [40]. To combine two primer datasets, ASV abundances were combined at the genus level, i.e., the highest possible taxonomic resolution shared between the datasets. Nonmetric multidimensional scaling (NMDS) was conducted using the Bray–Curtis distance. Permutational multivariate analysis of variance (PERMANOVA) was performed to disentangle variation in microbiota compositions by ADONIS. A complementary multivariate homogeneity of group dispersions (variances) analysis (betadisper) showed no dispersion effect between primers in community profiling (*p* > 0.05, betadisper test), confirming compositional dissimilarity in ADONIS results. The Procrustes analysis and Mantel test were performed to confirm the correlation between the community compositions characterized by different primers.

Species abundance distribution (SAD) analysis was conducted using the mean relative abundance and prevalence, the percentage of communities in which a given ASV is present [41]. The divergence of SAD between primer sets was assessed using two-dimensional Kullback–Leibler Divergence (KLD). The lower the sequence detection rate, the flatter the curve of SAD is. The detection rate is affected by the amplification efficiency, including overall and species-specific amplification efficiency. The differences in sequencing depth may intensify primer biases resulting from amplification efficiency, thereby contributing to the divergence in SAD. Species with higher amplification efficiency are more likely to be detected in low sequencing depth [42,43]. By doing *in silico*-down sampling, low sequencing depth could simulate the effect of amplification efficiency. To simulate the effect of amplification efficiency, down sampling was performed in two primer datasets with the same parameters at a depth of 1000.

To reveal the primer bias on habitat niche characterization, we applied Levins’ niche breadth index (*B*) and Morisita-Horn’s niche overlap (*O*) index [44−46] described by the formulas:

$$B_{j}=1/{\sum_{i=1}^{N} P_{ij}^{2}} ,$$

where *B_j_* indicates niche breadth, and *P_ij_* is the proportion of individuals belonging to species *j* present in a given habitat *i*.

$$O_{jk}=\frac{2\sum_{i=1}^{N} P_{ij}P_{ik}}{\sum_{i=1}^{N} P_{ij}^{2}+\sum_{i=1}^{N} P_{ik}^{2}} ,$$

where $O_{jk}$ indicates the niche overlap strength between two species *j* and *k* amongst all *N* habitats, and *P_ij_* or *P_ik_* is the proportion of species *j* or *k*, respectively, in a specific habitat.

*B*com (community-level niche breadth, the average of *B*-values from all taxa within one community) and *O*com (community-level niche overlap, the average of *O*-values from all taxon pairs within the community) were calculated. Microbial communities with a broader niche breadth are expected to be more metabolically flexible at the community level [47]. Microbial communities with a strengthened niche overlap are expected to have higher competition at the community level due to the large proportion of neutral or negative interactions amongst microorganisms [48,49].

Network analysis was performed to explore co-occurrence patterns as previously described [50]. To reduce rare OTUs in the dataset, we removed OTUs with a relative abundance <0.01%. Robust correlations with Spearman’s correlation coefficients (ρ) > 0.6 and p-values <0.01 were used to construct networks. To describe the topology of the networks, we calculated a set of metrics: average degree, average path length, clustering coefficient, network diameter, graph density, and modularity. The checkerboard score (C-score) was calculated to evaluate the real distributions for the non-randomness of OTUs by examining the deviation of each observed metric from the average of the null model [51]. The values obtained were standardized to allow comparisons amongst communities using the standardized effect size (SES).

# References

1. FAO. last accessed). Zhejiang Huzhou Mulberry-dyke & Fish-pond System, China. <https://www.fao.org/giahs/giahsaroundtheworld/designated-sites/asia-and-the-pacific/huzhou-mulberry/en/>.

2. Chen, Bosheng, Kaiqian Du, Chao Sun, Arunprasanna Vimalanathan, Xili Liang, Yong Li, Baohong Wang, Xingmeng Lu, Lanjuan Li, Yongqi Shao. 2018. "Gut bacterial and fungal communities of the domesticated silkworm (Bombyx mori) and wild mulberry-feeding relatives." *The ISME Journal* 12: 2252−2262. <https://doi.org/10.1038/s41396-018-0174-1>

3. Gilbert, Jack A., Janet K. Jansson, Rob Knight. 2014. "The Earth Microbiome project: successes and aspirations." *BMC Biology* 12: 69. <https://doi.org/10.1186/s12915-014-0069-1>

4. Liu, Shaohui, Qingwen Min, Wenjun Jiao, Chuanjiang Liu, Jianzhong Yin. 2018. "Integrated Emergy and Economic Evaluation of Huzhou Mulberry-Dyke and Fish-Pond Systems." *Sustainability* 10: 3860. <https://doi.org/10.3390/su10113860>

5. Yuan, Julin, Meng Ni, Mei Liu, Haiyang Wang, Chao Zhang, Guoqiang Mi, Zhimin Gu. 2019. "Analysis of the growth performances, muscle quality, blood biochemistry and antioxidant status of Micropterus salmoides farmed in in-pond raceway systems versus usual-pond systems." *Aquaculture* 511: 734241. <https://doi.org/10.1016/j.aquaculture.2019.734241>

6. Behera, Pratiksha, Sofia Mahapatra, Madhusmita Mohapatra, Ji Yoon Kim, Tapan K. Adhya, Vishakha Raina, Mrutyunjay Suar, Ajit K. Pattnaik, Gurdeep Rastogi. 2017. "Salinity and macrophyte drive the biogeography of the sedimentary bacterial communities in a brackish water tropical coastal lagoon." *Science of The Total Environment* 595: 472−485. <https://doi.org/10.1016/j.scitotenv.2017.03.271>

7. Jiao, Shuo, Baogang Zhang, Guozhuang Zhang, Weimin Chen, Gehong Wei. 2021. "Stochastic community assembly decreases soil fungal richness in arid ecosystems." *Molecular Ecology* 30: 4338−4348. <https://doi.org/10.1111/mec.16047>

8. Zhang, Nan, Jintao He, Xiaoqiang Shen, Chao Sun, Abrar Muhammad, Yongqi Shao. 2021. "Contribution of sample processing to gut microbiome analysis in the model Lepidoptera, silkworm Bombyx mori." *Computational and Structural Biotechnology Journal* 19: 4658−4668. <https://doi.org/10.1016/j.csbj.2021.08.020>

9. He, Jintao, Nan Zhang, Xiaoqiang Shen, Abrar Muhammad, Yongqi Shao. 2022. "Deciphering environmental resistome and mobilome risks on the stone monument: A reservoir of antimicrobial resistance genes." *Science of The Total Environment* 838: 156443. <https://doi.org/10.1016/j.scitotenv.2022.156443>

10. Bai, Yang, Daniel B. Müller, Girish Srinivas, Ruben Garrido-Oter, Eva Potthoff, Matthias Rott, Nina Dombrowski, et al. 2015. "Functional overlap of the Arabidopsis leaf and root microbiota." *Nature* 528: 364−369. <https://doi.org/10.1038/nature16192>

11. Parada, Alma E., David M. Needham, Jed A. Fuhrman. 2016. "Every base matters: assessing small subunit rRNA primers for marine microbiomes with mock communities, time series and global field samples." *Environmental Microbiology* 18: 1403−1414. <https://doi.org/10.1111/1462-2920.13023>

12. Fitzpatrick, Connor R., Patricia Lu-Irving, Julia Copeland, David S. Guttman, Pauline W. Wang, David A. Baltrus, Katrina M. Dlugosch, Marc T. J. Johnson. 2018. "Chloroplast sequence variation and the efficacy of peptide nucleic acids for blocking host amplification in plant microbiome studies." *Microbiome* 6: 144. <https://doi.org/10.1186/s40168-018-0534-0>

13. Martin, Marcel. 2011. "Cutadapt removes adapter sequences from high-throughput sequencing reads." *EMBnet.journal* 17: 10−12. <https://doi.org/10.14806/ej.17.1.200>

14. Zhang, Jiajie, Kassian Kobert, Tomáš Flouri, Alexandros Stamatakis. 2014. "PEAR: a fast and accurate Illumina Paired-End reAd mergeR." *Bioinformatics* 30: 614−620. <https://doi.org/10.1093/bioinformatics/btt593>

15. Callahan, Benjamin J., Paul J. McMurdie, Michael J. Rosen, Andrew W. Han, Amy Jo A. Johnson, Susan P. Holmes. 2016. "DADA2: High-resolution sample inference from Illumina amplicon data." *Nature Methods* 13: 581−583. <https://doi.org/10.1038/nmeth.3869>

16. Bokulich, Nicholas A., Benjamin D. Kaehler, Jai Ram Rideout, Matthew Dillon, Evan Bolyen, Rob Knight, Gavin A. Huttley, J. Gregory Caporaso. 2018. "Optimizing taxonomic classification of marker-gene amplicon sequences with QIIME 2’s q2-feature-classifier plugin." *Microbiome* 6: 90. <https://doi.org/10.1186/s40168-018-0470-z>

17. Davis, Nicole M., Diana M. Proctor, Susan P. Holmes, David A. Relman, Benjamin J. Callahan. 2018. "Simple statistical identification and removal of contaminant sequences in marker-gene and metagenomics data." *Microbiome* 6: 226. <https://doi.org/10.1186/s40168-018-0605-2>

18. Wemheuer, Franziska, Jessica A. Taylor, Rolf Daniel, Emma Johnston, Peter Meinicke, Torsten Thomas, Bernd Wemheuer. 2020. "Tax4Fun2: prediction of habitat-specific functional profiles and functional redundancy based on 16S rRNA gene sequences." *Environmental Microbiome* 15: 11. <https://doi.org/10.1186/s40793-020-00358-7>

19. Douglas, Gavin M., Vincent J. Maffei, Jesse R. Zaneveld, Svetlana N. Yurgel, James R. Brown, Christopher M. Taylor, Curtis Huttenhower, Morgan G. I. Langille. 2020. "PICRUSt2 for prediction of metagenome functions." *Nature Biotechnology* 38: 685−688. <https://doi.org/10.1038/s41587-020-0548-6>

20. Louca, Stilianos, Laura Wegener Parfrey, Michael Doebeli. 2016. "Decoupling function and taxonomy in the global ocean microbiome." *Science* 353: 1272−1277. <https://doi.org/10.1126/science.aaf4507>

21. Tian, Liang, Xu-Wen Wang, Ang-Kun Wu, Yuhang Fan, Jonathan Friedman, Amber Dahlin, Matthew K. Waldor, George M. Weinstock, Scott T. Weiss, Yang-Yu Liu. 2020. "Deciphering functional redundancy in the human microbiome." *Nature Communications* 11: 6217. <https://doi.org/10.1038/s41467-020-19940-1>

22. Legendre, Pierre. 2014. "Interpreting the replacement and richness difference components of beta diversity." *Global Ecology and Biogeography* 23: 1324−1334. <https://doi.org/10.1111/geb.12207>

23. Schmera, Dénes, János Podani, Pierre Legendre. 2020. "What do beta diversity components reveal from presence-absence community data? Let us connect every indicator to an indicandum!" *Ecological Indicators* 117: 106540. <https://doi.org/10.1016/j.ecolind.2020.106540>

24. Podani, János, Dénes Schmera. 2011. "A new conceptual and methodological framework for exploring and explaining pattern in presence – absence data." *Oikos* 120: 1625−1638. <https://doi.org/10.1111/j.1600-0706.2011.19451.x>

25. Baselga, Andrés. 2010. "Partitioning the turnover and nestedness components of beta diversity." *Global Ecology and Biogeography* 19: 134−143. <https://doi.org/10.1111/j.1466-8238.2009.00490.x>

26. Si, Xingfeng, Yuhao Zhao, Chuanwu Chen, Peng Ren, Di Zeng, Lingbing Wu, Ping Ding. 2017. "Beta-diversity partitioning: methods, applications and perspectives." *Biodiversity Science* 25: 464.

27. Shi, Yu, Yuntao Li, Xingjia Xiang, Ruibo Sun, Teng Yang, Dan He, Kaoping Zhang, et al. 2018. "Spatial scale affects the relative role of stochasticity versus determinism in soil bacterial communities in wheat fields across the North China Plain." *Microbiome* 6: 27. <https://doi.org/10.1186/s40168-018-0409-4>

28. Stegen, James C., Xueju Lin, Jim K. Fredrickson, Xingyuan Chen, David W. Kennedy, Christopher J. Murray, Mark L. Rockhold, Allan Konopka. 2013. "Quantifying community assembly processes and identifying features that impose them." *The ISME Journal* 7: 2069−2079. <https://doi.org/10.1038/ismej.2013.93>

29. Ning, Daliang, Ye Deng, James M. Tiedje, Jizhong Zhou. 2019. "A general framework for quantitatively assessing ecological stochasticity." *Proceedings of the National Academy of Sciences* 116: 16892−16898. <https://doi.org/10.1073/pnas.1904623116>

30. Sloan, William T., Mary Lunn, Stephen Woodcock, Ian M. Head, Sean Nee, Thomas P. Curtis. 2006. "Quantifying the roles of immigration and chance in shaping prokaryote community structure." *Environmental Microbiology* 8: 732−740. <https://doi.org/10.1111/j.1462-2920.2005.00956.x>

31. Stegen, James C., Xueju Lin, Allan E. Konopka, James K. Fredrickson. 2012. "Stochastic and deterministic assembly processes in subsurface microbial communities." *The ISME Journal* 6: 1653−1664. <https://doi.org/10.1038/ismej.2012.22>

32. Stegen, James C., Xueju Lin, Jim K. Fredrickson, Allan E. Konopka. 2015. "Estimating and mapping ecological processes influencing microbial community assembly." *Frontiers in Microbiology* 6: 370. <https://doi.org/10.3389/fmicb.2015.00370>

33. Pan, Yongbo, Jun Yang, George B. McManus, Senjie Lin, Wenjing Zhang. 2020. "Insights into protist diversity and biogeography in intertidal sediments sampled across a range of spatial scales." *Limnology and Oceanography* 65: 1103−1115. <https://doi.org/10.1002/lno.11375>

34. Wear, Emma K., Elizabeth G. Wilbanks, Craig E. Nelson, Craig A. Carlson. 2018. "Primer selection impacts specific population abundances but not community dynamics in a monthly time-series 16S rRNA gene amplicon analysis of coastal marine bacterioplankton." *Environmental Microbiology* 20: 2709−2726. <https://doi.org/10.1111/1462-2920.14091>

35. Mod, Heidi K., Aline Buri, Erika Yashiro, Nicolas Guex, Lucie Malard, Eric Pinto-Figueroa, Marco Pagni, Hélène Niculita-Hirzel, Jan Roelof van der Meer, Antoine Guisan. 2021. "Predicting spatial patterns of soil bacteria under current and future environmental conditions." *The ISME Journal* 15: 2547−2560. <https://doi.org/10.1038/s41396-021-00947-5>

36. Yilmaz, Pelin, Laura Wegener Parfrey, Pablo Yarza, Jan Gerken, Elmar Pruesse, Christian Quast, Timmy Schweer, Jörg Peplies, Wolfgang Ludwig, Frank Oliver Glöckner. 2014. "The SILVA and “All-species Living Tree Project (LTP)” taxonomic frameworks." *Nucleic Acids Research* 42: D643−D648. <https://doi.org/10.1093/nar/gkt1209>

37. McDonald, Daniel, Yueyu Jiang, Metin Balaban, Kalen Cantrell, Qiyun Zhu, Antonio Gonzalez, James T. Morton, et al. 2023. "Greengenes2 unifies microbial data in a single reference tree." *Nature Biotechnology* <https://doi.org/10.1038/s41587-023-01845-1>

38. Chao, Anne, Chun-Huo Chiu, Lou Jost. 2014. "Unifying Species Diversity, Phylogenetic Diversity, Functional Diversity, and Related Similarity and Differentiation Measures Through Hill Numbers." *Annual Review of Ecology, Evolution, and Systematics* 45: 297−324. <https://doi.org/10.1146/annurev-ecolsys-120213-091540>

39. Klindworth, Anna, Elmar Pruesse, Timmy Schweer, Jörg Peplies, Christian Quast, Matthias Horn, Frank Oliver Glöckner. 2013. "Evaluation of general 16S ribosomal RNA gene PCR primers for classical and next-generation sequencing-based diversity studies." *Nucleic Acids Research* 41: e1. <https://doi.org/10.1093/nar/gks808>

40. McCarthy, Davis J., Yunshun Chen, Gordon K. Smyth. 2012. "Differential expression analysis of multifactor RNA-Seq experiments with respect to biological variation." *Nucleic Acids Research* 40: 4288−4297. <https://doi.org/10.1093/nar/gks042>

41. Matthews, Thomas J., Robert J. Whittaker. 2015. "REVIEW: On the species abundance distribution in applied ecology and biodiversity management." *Journal of Applied Ecology* 52: 443−454. <https://doi.org/10.1111/1365-2664.12380>

42. O’Donnell, James L., Ryan P. Kelly, Natalie C. Lowell, Jesse A. Port. 2016. "Indexed PCR Primers Induce Template-Specific Bias in Large-Scale DNA Sequencing Studies." *PLOS ONE* 11: e0148698. <https://doi.org/10.1371/journal.pone.0148698>

43. Kelly, Ryan P., Andrew Olaf Shelton, Ramón Gallego. 2019. "Understanding PCR Processes to Draw Meaningful Conclusions from Environmental DNA Studies." *Scientific Reports* 9: 12133. <https://doi.org/10.1038/s41598-019-48546-x>

44. Levins, Richard. 1968. Evolution in changing environments: some theoretical explorations. *Princeton University Press*

45. Horn, S. Henry. 1966. "Measurement of Overlap in Comparative Ecological Studies." *American Naturalist* 100: 419−&. <https://doi.org/10.1086/282436>

46. Morisita, Masaaki. 1959. "Measuring of interspecific association and similarity between communities." *Memoirs Faculty of Science, Kyushu University. Series E (Biol.)* 3: 65−80.

47. Wu, Wenxue, Hsiao-Pei Lu, Akash Sastri, Yi-Chun Yeh, Gwo-Ching Gong, Wen-Chen Chou, Chih-Hao Hsieh. 2018. "Contrasting the relative importance of species sorting and dispersal limitation in shaping marine bacterial versus protist communities." *The ISME Journal* 12: 485−494. <https://doi.org/10.1038/ismej.2017.183>

48. Foster, Kevin R, Thomas Bell. 2012. "Competition, Not Cooperation, Dominates Interactions among Culturable Microbial Species." *Current Biology* 22: 1845−1850. <https://doi.org/10.1016/j.cub.2012.08.005>

49. He, Jintao, Nan Zhang, Abrar Muhammad, Xiaoqiang Shen, Chao Sun, Qiang Li, Yulan Hu, Yongqi Shao. 2022. "From surviving to thriving, the assembly processes of microbial communities in stone biodeterioration: A case study of the West Lake UNESCO World Heritage area in China." *Science of The Total Environment* 805: 150395. <https://doi.org/10.1016/j.scitotenv.2021.150395>

50. Jiao, Shuo, Yunfeng Yang, Yiqin Xu, Jie Zhang, Yahai Lu. 2020. "Balance between community assembly processes mediates species coexistence in agricultural soil microbiomes across eastern China." *The ISME Journal* 14: 202−216. <https://doi.org/10.1038/s41396-019-0522-9>

51. Gotelli, Nicholas J., Declan J. McCabe. 2002. "SPECIES CO-OCCURRENCE: A META-ANALYSIS OF J. M. DIAMOND'S ASSEMBLY RULES MODEL." *Ecology* 83: 2091−2096. [https://doi.org/10.1890/0012-9658(2002)083[2091:SCOAMA]2.0.CO;2](https://doi.org/10.1890/0012-9658(2002)083%5b2091:SCOAMA%5d2.0.CO;2)
